# Clinicopathological and Prognostic Significance of CD24 Overexpression in Patients with Gastric Cancer: A Meta-Analysis

**DOI:** 10.1371/journal.pone.0114746

**Published:** 2014-12-12

**Authors:** Jing-Xun Wu, Yuan-Yuan Zhao, Xuan Wu, Han-Xiang An

**Affiliations:** 1 Department of Medical Oncology, The First Affiliated Hospital of Xiamen University, Xiamen, Fujian, China; 2 State Key Laboratory of Oncology in South China, Department of Medical Oncology, Sun Yat-Sen University Cancer Center, Guangzhou, Guangdong, China; 3 Department of Medical Oncology, Peking University Shenzhen Hospital, Shenzhen, Guangdong, China; Nanjing, China

## Abstract

**Objective:**

The prognostic significance of CD24 expression for survival in patients with gastric cancer remains controversial. We conducted a meta-analysis to investigate the impact of CD24 expression on clinicopathological features and survival outcomes in gastric cancer.

**Methods:**

A comprehensive literature search of the electronic databases PubMed, Embase, Web of Science and China National Knowledge Infrastructure (CNKI; up to April 8, 2014) was performed for relevant studies using multiple search strategies. Correlations between CD24 expression and clinicopathological features and overall survival (OS) were analyzed.

**Results:**

A total of 1,041 patients with gastric cancer from 9 studies were included. The pooled odds ratios (ORs) indicated CD24 expression was associated with tumor depth (OR = 0.45, 95% confidence interval [CI]  = 0.32–0.63; P<0.00001), status of lymph nodes (OR = 0.40, 95% CI = 0.25–0.64; P = 0.0001) and tumor node metastasis (TNM) stage (OR = 0.56, 95% CI = 0.41–0.77; P = 0.0003). The pooled hazard ratio (HR) for OS showed overexpression of CD24 reduced OS in gastric cancer (HR = 1.99, 95% CI = 1.29–3.07, P = 0.002). Whereas, combined ORs showed that CD24 expression had no correlation with tumor differentiation or Lauren classifications.

**Conclusion:**

CD24 overexpression in patients with gastric cancer indicated worse survival outcomes and was associated with common clinicopathological poor prognostic factors.

## Introduction

Gastric cancer (GC) is the fourth most common cancer and the second leading cause of cancer-related death worldwide [1]. Although having undergone radical resection and postoperative adjuvant therapy, most patients with GC will die of recurrence and metastasis. Several clinicopathological parameters such as tumor size, histological type, tumor differentiation, depth of tumor invasion, regional lymph node involvement, distant metastasis and tumor stage, have been reported as important prognostic factors for GC [2]–[6]. However, the advance in treatment of GC was relatively small in the past few decades. Understanding the molecular mechanisms that lead to the development and progression of GC remains an important challenge in translational research.

CD24, a glycosylphosphatidylinositol (GPI)-anchored membrane protein, is a ligand of P-selectin, which is an adhesive molecule on activated endothelial cells and platelets [7]–[10]. CD24 is expressed by B lymphocytes, T cells, neutrophils, neuronal tissue, keratinocytes and renal tubular epithelial cells [11]–[13]. Several studies have shown that CD24 played important roles in the regulation of B-cell apoptosis, leukocyte signal transduction, leukocyte adhesion and cell selection or maturation during hematopoiesis [8]–[10], [14], [15].

Using immunohistochemistry, a series of studies has revealed that CD24 was expressed in a variety of human malignancies, such as nasopharyngeal carcinoma, non-small-cell lung cancer, breast cancer, hepatocellular carcinoma, pancreatic cancer, colorectal cancer, renal cell carcinoma, bladder carcinoma, ovarian cancer, prostate cancer and intrahepatic cholangiocarcinoma [16], [17]. Expression of CD24 might facilitate the interactions of cancer cells with endothelial cells and platelets, which promotes the dissemination of CD24-expressing cancer cells [18], [19]. Previous studies have shown that the expression of CD24 is correlated with tumor progression and a poor prognosis in various carcinomas [20].

Although evidence exists that CD24 is an important factor implicated in clinicopathological features [21]–[29] and the prognosis of GC [22], [23], some conflicting results have been reported. A study on CD24 in GC reported that positive CD24 expression tended to indicate worse survival outcomes, but the difference was not statistically significant [27]. Moreover, two other recent studies on a panel of tumor markers demonstrated that CD24 expression was not an independent prognostic factor for patients with GC [25], [28]. Whether discrepancy in these data was due to limited sample sizes or genuine heterogeneity is still unknown. To address the controversial issues, a meta-analysis was carried out to determine the association between CD24 and clinicopathological parameters as well as the significance of CD24 expression in the prediction of clinical outcomes in GC.

## Materials and Methods

### Search Strategy

A comprehensive literature search of the electronic databases PubMed, Embase, Web of Science and China National Knowledge Infrastructure (CNKI) was performed up to April 8, 2014. Studies were selected using the following search terms: ‘gastric or stomach’ and ‘cancer or neoplasm or carcinoma’ and ‘CD24’. The references of articles and reviews were also manually searched for additional studies. The eligible reports were identified by two reviewers (J-X.W. and Y-Y.Z.) and controversial studies were adjudicated by a third reviewer (H-X.A.).

### Study Selection

We collected all eligible articles about the relationship between CD24 and clinicopathological features and clinical outcomes in GC in this meta-analysis. Studies meeting the following inclusion criteria were included: (1) CD24 expression was evaluated in the primary GC tissues; (2) CD24 expression was examined by immunohistochemistry (IHC); (3) studies revealed the relationship between CD24 expression and GC clinicopathological parameters or prognosis; (4) studies regarding the prognosis provided sufficient information to estimate hazard ratios (HRs) for overall survival (OS) and 95% confidenceintervals (CIs); (5) if there were multiple articles based on similar patients, only the largest or most recently published article was included. The exclusion criteria used in this meta-analysis were: (1) letters, reviews, case reports, conference abstracts, editorials and expert opinion; and (2) patients who had received previous chemotherapy or radiotherapy.

### Data Extraction

Two investigators (J-X.W. and H-X.A.) independently extracted data from eligible studies. Disagreements were resolved by discussion and consensus. Two investigators reviewed all studies that met the inclusion and exclusion criteria. The following information was recorded for each study: name of the first author, year of publication, sample source, number of cases, clinicopathological parameters, Lauren classification for gastric adenocarcinomas [30], tumor node metastasis (TNM) stage, immunohistochemical technique, definition of a positive CD24, and survival of patients. If the HR or standard errors (SEs) were not reported in the included studies, we calculated or estimated the HR from available data or Kaplan-Meier curves using the methods reported by Tierney et al. [31].

### Assessment of Study Quality

Two authors (J-X.W. and Y-Y.Z.) independently assessed the quality of all studies on the basis of a 9-score system; i.e., the Newcastle-Ottawa Scale (NOS) [32]. In this scoring system, each study included in the meta-analysis was judged on three broad perspectives: the selection of the study cases (four items, one score for each item), the comparability of the study populations (one item, up to two scores) and the ascertainment of either the exposure or outcome of interest (three items, one score for each item). Scores of items were added up and used to compare study quality in a quantitative manner. A higher score indicated the individual study was of higher quality. Discrepancies in the score were resolved through discussion between the authors.

### Statistical Methods

The meta-analysis was performed using the Stata 12.0 (Stata Corporation, College Station, TX, USA) and Review Manager 5.2 (Cochrane Collaboration, Oxford, UK) programs. Comparisons of CD24 expression in groups with different clinicopathologic features were performed by pooled estimates of odds ratios (ORs), as well as the 95% CI. Statistical significance was determined at α = 0.05. In consideration of the possibility of heterogeneity among the studies, a statistical test for heterogeneity was examined by the Chi-square-based Q-test, and the significance level was fixed at P<0.10. The inconsistency index I^2^ was also calculated to evaluate the variation caused by heterogeneity. A high value of I^2^ indicated a higher probability of the existence of heterogeneity. The DerSimonian and Laird random-effects model [33] was used if substantial heterogeneity was detected (Q-statistic: P<0.10; I^2^>50%). Otherwise, a fixed-effects model of Mantel-Haenszel [34] was applied in the absence of between-study heterogeneity (Q-statistic: P>0.10; I^2^<50%). Publication bias was assessed by a Begg's rank correlation test and Egger's regression asymmetry test [35], [36].

## Results

### Search Results

**Figure 1:**
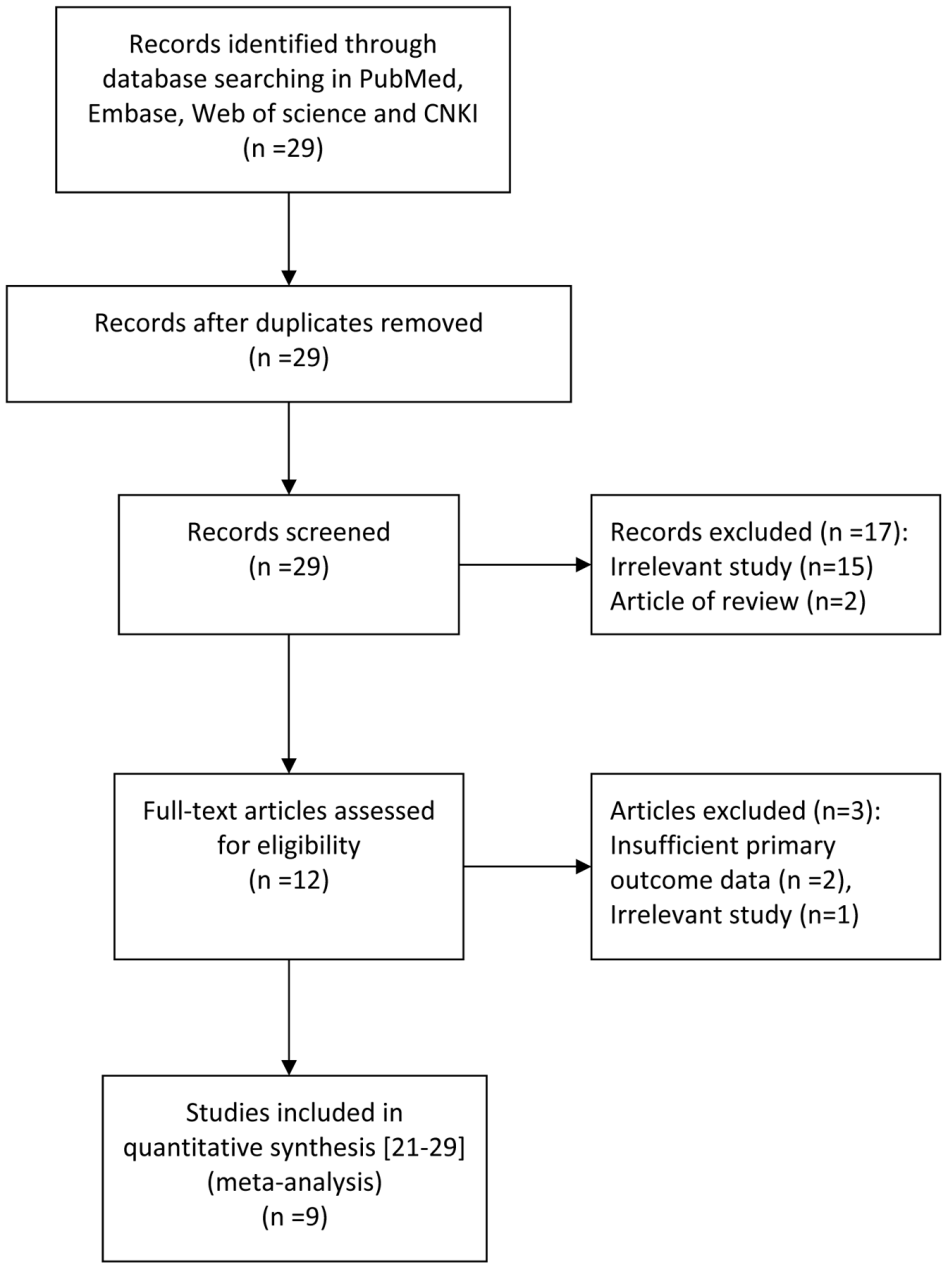
Flow chart for selection of studies. Excluded studies with the reasons for exclusion were listed in S1 Table.

Twenty-nine articles were identified using the search strategy described above. Twenty of them were excluded due to being irrelevant to the current analysis, non-original articles (review) or having insufficient primary outcome data. There were nine studies finally included in the current meta-analysis (Fig. 1).

### Study Characteristics

Nine publications from 2004 to 2014 were eligible for meta-analysis. Their characteristics are summarized in Table 1. A total of 1,041 patients from China, Korea, Turkey and Japan were enrolled, including 707 males and 334 females. CD24 overexpression was found in 521 patients (50.0%). Immunohistochemistry was the only method used to evaluate the expression of CD24 in GC specimens. The definition of CD24 positive staining varied among the studies. Six researches defined CD24 expression by different percentages of positive cells, whereas only one study determined CD24 expression by both staining intensity score and percentage of positive cells.

**Table 1:** Characteristics of eligible studies.

First Author	Year	Origin	Cases	Method	Antibody source	Dilution	CD24 distribution	Counting method	Definition of CD24 positive	Scores of study quality
Darwish [23]	2004	Korea	300	IHC	Neomarkers	-	Membrane and cytoplasma	-	-	6
Chou [22]	2007	Taiwan	103	IHC	Neomarkers	1∶100	Cytoplasma	Percentage of positive cells	>0%	8
Yao [29]	2008	China	49	IHC	MAB	-	Membrane and cytoplasma	Percentage of positive cells	≥25%	6
Jian [24]	2009	China	56	IHC	MAB	1∶100	Membrane and cytoplasma	Percentage of positive cells	>0%	7
Niu [26]	2009	China	68	IHC	Bioss	-	Cytoplasma	Percentage of positive cells	≥10%	6
Bektas [21]	2010	Turkey	93	IHC	Neomarkers	1∶50	Cytoplasma	Percentage of positive cells	>0%	7
Yang [28]	2012	China	95	IHC	Abgent	-	Membrane and cytoplasma	-	-	5
Takahashi [27]	2013	Japan	173	IHC	Thermo	-	Membrane and cytoplasma	Percentage of positive cells	≥10%	7
Liu [25]	2014	China	104	IHC	Dako	-	Cytoplasma	staining intensity score and percentage of positive cells	≥1.0	7

*IHC* Immunohistochemistry.

### Qualitative Assessment

The study quality was assessed using the Newcastle-Ottawa quality assessment scale, generating scores ranging from 5 to 8 (with a mean of 6.56), with a higher value indicating better methodology. The results of quality assessment are shown in Table 1.

### Quantitative Synthesis

#### Correlation of CD24 expression and clinicopathological features

**Figure 2:**
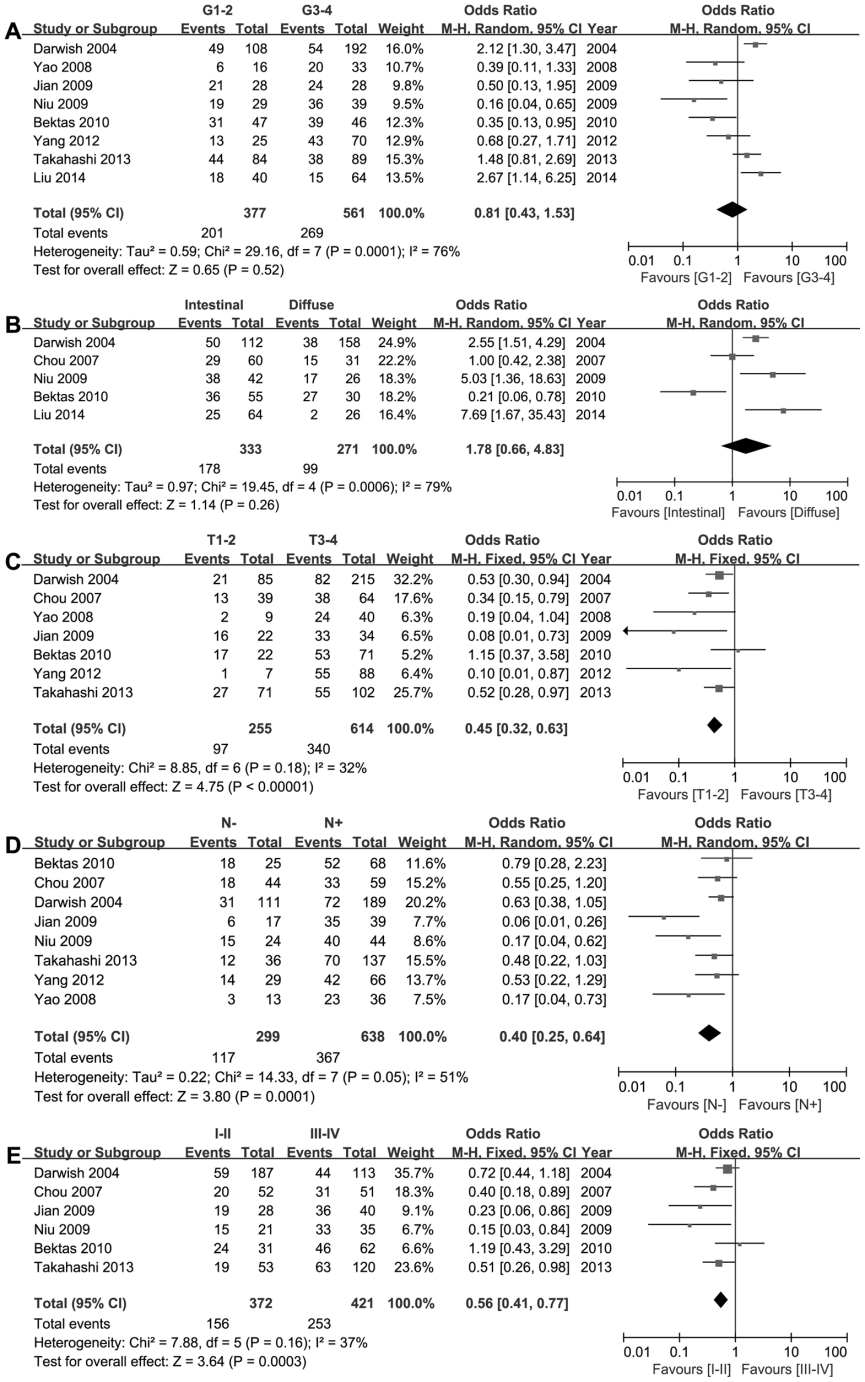
Forest plots of CD24 expression and the clinicopathological features of patients with gastric cancer. The squares and horizontal lines correspond to the study- specific OR and 95% CI. The area of the squares reflects the study-specific weight (inverse of the variance). The diamonds represent the pooled OR and 95% CI. The solid vertical line is at the null value (OR = 1). **A The relationship between CD24 expression and tumor differentiation.** CD24 expression was not related to tumor differentiation (OR = 0.81, 95% CI = 0.43–1.53, P = 0.52). **B The relationship between CD24 expression and Lauren classification.** CD24 expression was not different in intestinal-type and diffuse-type of gastric adenocarcinoma. (OR = 1.78, 95% CI = 0.66–4.83, P = 0.26). **C The relationship between CD24 expression and tumor depth.** CD24 expression was associated with tumor depth (OR = 0.45, 95% CI = 0.32–0.63; P<0.00001). **D The relationship between CD24 expression and status of lymph node.** CD24 expression was associated with status of lymph node (OR = 0.40, 95% CI = 0.25–0.64; P = 0.0001). **E The relationship between CD24 expression and TNM staging.** CD24 expression was associated with TNM staging of gastric cancer (OR = 0.56, 95% CI = 0.41–0.77; P = 0.0003). *G* grade; *T* tumor; *N* node.

The association between CD24 and histological differentiation was investigated in eight studies. The outcomes were significantly heterogeneous (P = 0.0001, I^2^ = 76%). Therefore, a random-effects model was used for the meta-analysis. The combined OR revealed CD24 expression was not related to tumor differentiation (OR = 0.81, 95% CI = 0.43–1.53, P = 0.52, Fig. 2A).

Five studies described CD24 expression according to Lauren classifications. It was found that CD24 expression was not different in intestinal-type and diffuse-type of gastric adenocarcinoma. (OR = 1.78, 95% CI = 0.66–4.83, P = 0.26, Fig. 2B). Significant heterogeneity was found among the studies (P = 0.0006, I^2^ = 79%).

Seven, eight and six of nine studies investigated the relationship of CD24 expression to invasive depth of tumor, status of lymph node metastasis and TNM stage, respectively. The results of the meta-analysis showed CD24 expression was associated with tumor depth (OR = 0.45, 95% CI = 0.32–0.63; P<0.00001, Fig. 2C), status of lymph nodes (OR = 0.40, 95% CI = 0.25–0.64; P = 0.0001, Fig. 2D) and TNM stage (OR = 0.56, 95% CI = 0.41–0.77; P = 0.0003, Fig. 2E). Heterogeneity was observed in the analysis of CD24 expression with status of lymph nodes (P = 0.05; I^2^ = 51%) and, therefore, a random-effects model was used.

#### CD24 as a prognostic factor for gastric cancer

**Figure 3:**
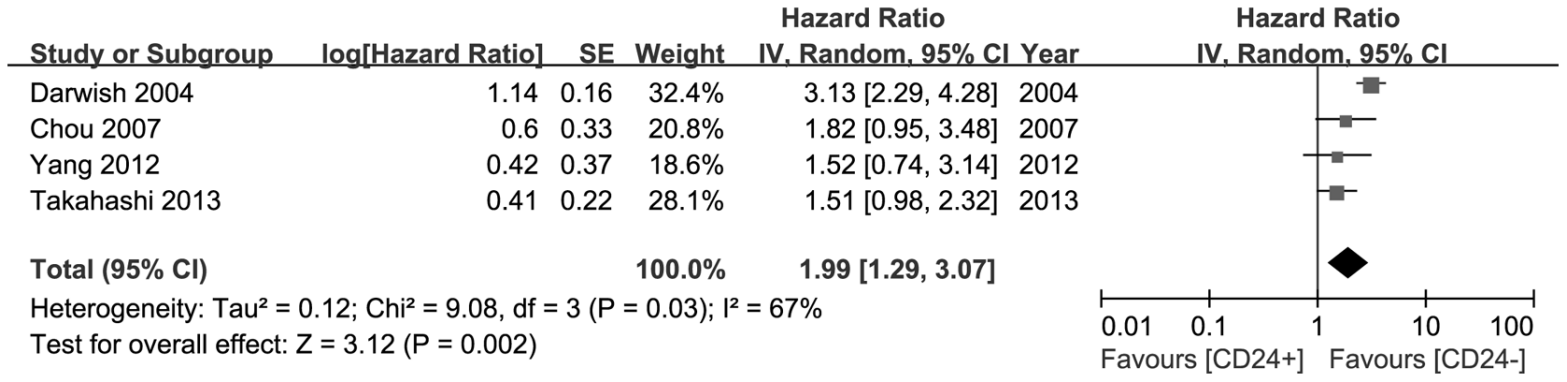
Forest plot of hazard ratio for overall survival of patients with gastric cancer. The squares and horizontal lines correspond to the study- specific HR and 95% CI. The area of the squares reflects the study-specific weight (inverse of the variance). The diamonds represent the pooled HR and 95% CI. The solid vertical line is at the null value (HR = 1). The pooled HR for OS showed overexpression of CD24 reduced OS in gastric cancer (HR = 1.99, 95%CI 1.29–3.07, P = 0.002).

Four of the nine studies included had estimated the relationship between OS and CD24 expression. Heterogeneity among the studies was statistically significant, so a random-effects model was used. The pooled HR for OS showed overexpression of CD24 was significantly associated with reduced OS in GC (HR = 1.99, 95%CI 1.29–3.07, P = 0.002, Fig. 3).

### Sensitivity Analyses and Publication Bias

A sensitivity analysis, in which one study was removed at a time, was performed to evaluate results stability. The corresponding pooled ORs and HRs were not significantly altered, suggesting stability of our results.

**Figure 4:**
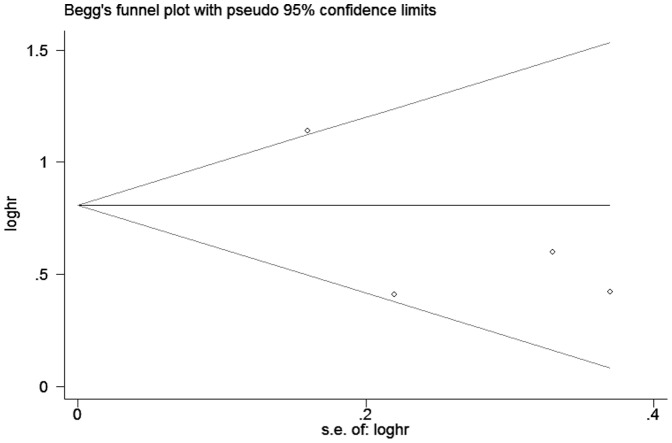
Begg's funnel plot of publication bias of hazard ratio for overall survival in the meta-analysis. The funnel plot displays log HR against its standard error (s.e.) for each individual study. The horizontal line represents the estimate of the overall HR, with the dash lines indicating the expected 95% CI for a given standard error. No evidence of publication bias was observed in the meta-analysis.

Egger's and Begg's tests indicated no publication bias among these studies regarding the HR for OS with P values of 0.272 and 0.734 respectively. The funnel plots were largely symmetric (Fig. 4), which also suggested no evidence of publication bias in this meta-analysis.

## Discussion

CD24 is a cell surface molecule that plays an important role in the migration and adhesion of cells [19]. The correlation between CD24 expression and GC has been studied by a number of researchers. Some of them showed overexpression of CD24 is associated with several clinicopathologic features and a poor prognosis [21]–[29]. However, the clinical relevance of CD24 as a prognostic factor in GC remains controversial. For procuring a reasonable conclusion, we combined 9 eligible studies including 1,041 cases to perform this meta-analysis.

The depth of tumor invasion, lymph node metastasis and distant metastases are the major prognostic factors in GC. It has been reported that CD24 expression increases the proliferation of tumor cells, induces cell motility and invasion and promotes cell spreading [11]. CD24 was identified as a ligand to P-selectin, which is a surface molecule expressed by activated endothelial cells and platelets. Tumor cells with increased CD24 expression acquired the capacity to form stabilized platelet-tumor thrombi and then adhered to endothelial cells at the distal metastatic sites, which protected tumor cells from destruction and promoted tissue penetration and tumor extravasation [11], [16], [22], [37]. On the other hand, by a subtractive technique, CD24 was revealed as one of the metastasis-associated genes and confirmed to be overexpressed in the metastatic phenotype [23]. A previous meta-analysis investigating the relationship between CD24 expression and prognostic parameters in different carcinomas suggested CD24 expression was associated with lymph node metastasis in breast cancer and advanced clinical stages in urothelial carcinomas. Limited by insufficient cases (151 patients were included), the expression of CD24 was found to be not associated with lymph node metastasis in GC [20]. In the present meta-analysis, we found CD24 expression was related to tumor depth, status of lymph nodes and TNM staging in an expanded sample. In line with basic studies, the results of our research supported that CD24 was involved in cell-cell and cell-matrix interactions. Although the mechanism of cancer progression caused by expressional alterations remains unknown, CD24 overexpression may be considered as a marker of GC that indicates invasiveness. Further functional investigation of CD24 activity in GC may clarify the importance of CD24 in the progression of the disease.

In previous studies, only one of four studies suggested statistically significant HRs for OS of elevated CD24 expression [23] and the remaining three studies showed a trend for reduced survival outcomes [22], [27], [28]. Sample size, as a strong predictor for epidemiological studies, may play an important role in this controversy. In the current meta-analysis, we pooled the data from these studies together, and demonstrated a remarkable association between CD24 expression and OS of patients with GC.

According to the Lauren classification system [30], gastric adenocarcinomas were classified into a diffuse or intestinal type. The diffuse type, which occurs more commonly in younger women, has a diffuse infiltrative growth pattern and is usually a poorly differentiated adenocarcinoma. In contrast, the intestinal type occurs more frequently in elderly men and is characterized by well-defined glandular structures and is always associated with atrophic gastritis and intestinal metaplasia in adjacent mucosa [38]. The diffuse type of GC has a familial tendency, relates to decreased E-cadherin, and has a less favorable prognosis, whereas the intestinal type is associated with environmental etiology, microsatellite instability and adenomatous polyposis coli (APC) mutation [39]. These findings indicated that the diffuse and intestinal typesof GC have distinct molecular pathways. Previous studies revealed there was a potential relationship between Lauren classifications and CD24 expression. However, our meta-analysis of these pooled patients, showed that overexpression of CD24 was not associated with the diffuse or intestinal types of GC.

Efforts were made to conduct a comprehensive analysis, but some limitations need to be acknowledged. First, the survival analysis was not performed by multivariate analyses in most reported studies. We, therefore, calculated the HR for OS from available data or Kaplan-Meier curves. Second, it was clear that the two types of GC might have different biological behaviors. Although our study demonstrated CD24 expression was not associated with Lauren classifications, whether CD24 played a different role in intestinal-type and diffuse-type GC is still unknown. Limited to insufficient information to estimate the relationship between CD24 and clinicopathologic features in different types of GC, more efforts are needed in the future. Third, the different concentrations of antibodies and the variable definitions of CD24 expression used in the included studies might have influenced the results of our meta-analysis. Fourth, most of the included studies were carried out in East Asia and the results may be different in Western countries.

Our meta-analysis indicated that overexpression of CD24 in GC was not only associated with tumor exterior expansion, lymph node metastasis and advanced TNM stage, but also was a biomarker for poor prognosis. Further clinical studies may be performed to clarify the precise prognostic significance of CD24 in GC.

## Supporting Information

S1 Table **Excluded studies and the reasons for exclusion.**
(DOC)

S1 Checklist **PRISMA checklist.**
(DOC)

## References

[1] Kamangar F, Dores GM, Anderson WF (2006) Patterns of cancer incidence, mortality, and prevalence across five continents: defining priorities to reduce cancer disparities in different geographic regions of the world. J Clin Oncol 24:2137–2150. 16682732 10.1200/JCO.2005.05.2308

[2] Adachi Y, Yasuda K, Inomata M, Sato K, Shiraishi N, et al (2000) Pathology and prognosis of gastric carcinoma: well versus poorly differentiated type. Cancer 89:1418–1424. 11013353

[3] Hundahl SA, Menck HR, Mansour EG, Winchester DP (1997) The National Cancer Data Base report on gastric carcinoma. Cancer 80:2333–2341. 9404711 10.1002/(sici)1097-0142(19971215)80:12<2333::aid-cncr15>3.0.co;2-v

[4] Maruyama K, Kaminishi M, Hayashi K, Isobe Y, Honda I, et al (2006) Gastric cancer treated in 1991 in Japan: data analysis of nationwide registry. Gastric Cancer 9:51–66. 16767357 10.1007/s10120-006-0370-y

[5] Serlin O, Keehn RJ, Higgins GA Jr, Harrower HW, Mendeloff GL (1977) Factors related to survival following resection for gastric carcinoma: analysis of 903 cases. Cancer 40:1318–1329. 143340 10.1002/1097-0142(197709)40:3<1318::aid-cncr2820400349>3.0.co;2-9

[6] Siewert JR, Bottcher K, Stein HJ, Roder JD (1998) Relevant prognostic factors in gastric cancer: ten-year results of the German Gastric Cancer Study. Annals of surgery 228:449–461. 9790335 10.1097/00000658-199810000-00002 PMC1191515

[7] Chen M, Geng JG (2006) P-selectin mediates adhesion of leukocytes, platelets, and cancer cells in inflammation, thrombosis, and cancer growth and metastasis. Archivum immunologiae et therapiae experimentalis 54:75–84. 16648968 10.1007/s00005-006-0010-6

[8] Fischer GF, Majdic O, Gadd S, Knapp W (1990) Signal transduction in lymphocytic and myeloid cells via CD24, a new member of phosphoinositol-anchored membrane molecules. J Immunol 144:638–641. 2153173

[9] Pirruccello SJ, LeBien TW (1986) The human B cell-associated antigen CD24 is a single chain sialoglycoprotein. J Immunol 136:3779–3784. 2939133

[10] Sammar M, Aigner S, Hubbe M, Schirrmacher V, Schachner M, et al (1994) Heat-stable antigen (CD24) as ligand for mouse P-selectin. International immunology 6:1027–1036. 7524641 10.1093/intimm/6.7.1027

[11] Baumann P, Cremers N, Kroese F, Orend G, Chiquet-Ehrismann R, et al (2005) CD24 expression causes the acquisition of multiple cellular properties associated with tumor growth and metastasis. Cancer research 65:10783–10793. 16322224 10.1158/0008-5472.CAN-05-0619

[12] Li O, Zheng P, Liu Y (2004) CD24 expression on T cells is required for optimal T cell proliferation in lymphopenic host. The Journal of experimental medicine 200:1083–1089. 15477346 10.1084/jem.20040779 PMC2211842

[13] Nielsen PJ, Lorenz B, Muller AM, Wenger RH, Brombacher F, et al (1997) Altered erythrocytes and a leaky block in B-cell development in CD24/HSA-deficient mice. Blood 89:1058–1067. 9028339

[14] Kadmon G, von Bohlen und Halbach F, Schachner M, Altevogt P (1994) Differential, LFA-1-sensitive effects of antibodies to nectadrin, the heat-stable antigen, on B lymphoblast aggregation and signal transduction. Biochemical and biophysical research communications 198:1209–1215. 8117278 10.1006/bbrc.1994.1171

[15] Suzuki T, Kiyokawa N, Taguchi T, Sekino T, Katagiri YU, et al (2001) CD24 induces apoptosis in human B cells via the glycolipid-enriched membrane domains/rafts-mediated signaling system. J Immunol 166:5567–5577. 11313396 10.4049/jimmunol.166.9.5567

[16] Kristiansen G, Sammar M, Altevogt P (2004) Tumour biological aspects of CD24, a mucin-like adhesion molecule. Journal of molecular histology 35:255–262. 15339045 10.1023/b:hijo.0000032357.16261.c5

[17] Su MC, Hsu C, Kao HL, Jeng YM (2006) CD24 expression is a prognostic factor in intrahepatic cholangiocarcinoma. Cancer letters 235:34–39. 16125303 10.1016/j.canlet.2005.03.059

[18] Aigner S, Ramos CL, Hafezi-Moghadam A, Lawrence MB, Friederichs J, et al (1998) CD24 mediates rolling of breast carcinoma cells on P-selectin. Faseb J 12:1241–1251. 9737727 10.1096/fasebj.12.12.1241

[19] Friederichs J, Zeller Y, Hafezi-Moghadam A, Grone HJ, Ley K, et al (2000) The CD24/P-selectin binding pathway initiates lung arrest of human A125 adenocarcinoma cells. Cancer research 60:6714–6722. 11118057

[20] Lee JH, Kim SH, Lee ES, Kim YS (2009) CD24 overexpression in cancer development and progression: a meta-analysis. Oncology reports 22:1149–1156. 19787233 10.3892/or_00000548

[21] Bektas S, Bahadir B, Ucan BH, Ozdamar SO (2010) CD24 and galectin-1 expressions in gastric adenocarcinoma and clinicopathologic significance. Pathol Oncol Res 16:569–577. 20177845 10.1007/s12253-010-9248-8

[22] Chou YY, Jeng YM, Lee TT, Hu FC, Kao HL, et al (2007) Cytoplasmic CD24 expression is a novel prognostic factor in diffuse-type gastric adenocarcinoma. Annals of surgical oncology 14:2748–2758. 17680316 10.1245/s10434-007-9501-x

[23] Darwish NS, Kim MA, Chang MS, Lee HS, Lee BL, et al (2004) Prognostic significance of CD24 expression in gastric carcinoma. Cancer Res Treat 36:298–302. 20368819 10.4143/crt.2004.36.5.298 PMC2843873

[24] Jian (2009) The expression and clinical significance of CD24 in human gastric carcinoma. Journal of Chongqing Medical University 34:1348–1350.

[25] Liu X, Yu H, Cai H, Wang Y (2014) Expression of CD24, p21, p53, and c-myc in alpha-fetoprotein-producing gastric cancer: Correlation with clinicopathologic characteristics and survival. Journal of surgical oncology 109:859–864. 24619835 10.1002/jso.23599

[26] Niu (2009) Expression and significaace of CD24 in precancerous lesion and gastric cancer. Journal of Nongken Medicine 31:129–132.

[27] Takahashi M, Nakajima M, Ogata H, Domeki Y, Ohtsuka K, et al (2013) CD24 expression is associated with progression of gastric cancer. Hepato-gastroenterology 60:653–658. 23159387 10.5754/hge12763

[28] Yang S-b, Xiao L-b, Feng-feng X (2012) Expression of EPCAM, CD44 and CD24 in gastric cancer tissues and its clinical significance. Chinese Journal of Pathophysiology 28:1224–1229.

[29] Yao H-l, YANG Z-l, LI Y-g, Wu W-h, Li S-z, et al (2008) Expression and significance of CD24 and CD44v6 in benign and malignant lesions of stomach. Chinese Journal of Pathophysiology 24:1629–1631.

[30] Lauren P (1965) The Two Histological Main Types of Gastric Carcinoma: Diffuse and So-Called Intestinal-Type Carcinoma. an Attempt at a Histo-Clinical Classification. Acta pathologica et microbiologica Scandinavica 64:31–49. 14320675 10.1111/apm.1965.64.1.31

[31] Tierney JF, Stewart LA, Ghersi D, Burdett S, Sydes MR (2007) Practical methods for incorporating summary time-to-event data into meta-analysis. Trials 8:16. 17555582 10.1186/1745-6215-8-16 PMC1920534

[32] Wells GA, Shea B, O'Connell D, Peterson J, Welch V, et al. (2011) The Newcatsle-Ottawa Scale (NOS) for assessing the quality of nonrandomised studies in meta-analysis. Ottawa: Ottawa Hospital Research Institute Available: www.ohri.ca/programs/clinical_epidemiology/oxford.htm accessed April 8, 2014.

[33] DerSimonian R, Laird N (1986) Meta-analysis in clinical trials. Controlled clinical trials 7:177–188. 3802833 10.1016/0197-2456(86)90046-2

[34] Mantel N, Haenszel W (1959) Statistical aspects of the analysis of data from retrospective studies of disease. Journal of the National Cancer Institute 22:719–748. 13655060

[35] Begg CB, Mazumdar M (1994) Operating characteristics of a rank correlation test for publication bias. Biometrics 50:1088–1101. 7786990

[36] Egger M, Davey Smith G, Schneider M, Minder C (1997) Bias in meta-analysis detected by a simple, graphical test. BMJ (Clinical research ed 315:629–634. 10.1136/bmj.315.7109.629 PMC2127453 9310563

[37] Aigner S, Sthoeger ZM, Fogel M, Weber E, Zarn J, et al (1997) CD24, a mucin-type glycoprotein, is a ligand for P-selectin on human tumor cells. Blood 89:3385–3395. 9129046

[38] Lynch HT, Grady W, Suriano G, Huntsman D (2005) Gastric cancer: new genetic developments. Journal of surgical oncology 90:114–133 discussion 133. 15895459 10.1002/jso.20214

[39] Ming SC (1998) Cellular and molecular pathology of gastric carcinoma and precursor lesions: A critical review. Gastric Cancer 1:31–50. 11957042 10.1007/s101200050053

