# Supplementary material for: Clinicopathological and Prognostic Significance of CD24 Overexpression in Patients with Gastric Cancer: A Meta-Analysis
Source: PLoS One. 2014 Dec 12;9(12):e114746. doi: 10.1371/journal.pone.0114746 (PMC4264770; doi:10.1371/journal.pone.0114746)
Supplement: S1 Table — Excluded studies and the reasons for exclusion. (DOC) [file pone.0114746.s001.doc]

Excluded studies and the reasons for exclusion

| No. | Excluded studies | Reasons for exclusion |
| --- | --- | --- |
| 1 | Wang YC, Wang JL, Kong X, Sun TT, Chen HY, Hong J, Fang JY. CD24 mediates gastric carcinogenesis and promotes gastric cancer progression via STAT3 activation. Apoptosis. 2014 Apr;19 (4):643-56. | Irrelevant study (Not relevant to the relationship between CD24 expression and gastric cancer clinicopathological parameters or prognosis) |
| 2 | Jiao XL, Zhao C, Niu M, Chen D. Downregulation of CD24 inhibits invasive growth, facilitates apoptosis and enhances chemosensitivity in gastric cancer AGS cells. Eur Rev Med Pharmacol Sci. 2013 Jul;17(13):1709-15. | Irrelevant study (Not relevant to the relationship between CD24 expression and gastric cancer clinicopathological parameters or prognosis) |
| 3 | Gao M, Cai Y, Wu W, Shi Y, Fei Z. A tissue-engineered gastric cancer model for mechanistic study of anti-tumor drugs. Biomed Mater. 2013 Aug;8 (4):045003. | Irrelevant study (Not relevant to CD 24) |
| 4 | Duckworth CA, Clyde D, Pritchard DM. CD24 is expressed in gastric parietal cells and regulates apoptosis and the response to Helicobacter felis infection in the murine stomach. Am J Physiol Gastrointest Liver Physiol. 2012 Oct 15;303(8):G915-26. | Irrelevant study (Not relevant to gastric cancer) |
| 5 | Ono Y, Hayashida T, Konagai A, Okazaki H, Miyao K, Kawachi S, Tanabe M, Shinoda M, Jinno H, Hasegawa H, Kitajima M, Kitagawa Y. Direct inhibition of the transforming growth factor-β pathway by protein-bound polysaccharide through inactivation of Smad2 signaling. Cancer Sci. 2012 Feb;103(2):317-24. | Irrelevant study (Not relevant to CD 24 and gastric cancer) |
| 6 | Zhang C, Li C, He F, Cai Y, Yang H. Identification of CD44+CD24+ gastric cancer stem cells. J Cancer Res Clin Oncol. 2011 Nov;137(11):1679-86. | Irrelevant study (Not relevant to the relationship between CD24 expression and gastric cancer clinicopathological parameters or prognosis) |
| 7 | Song Z, Yue W, Wei B, Wang N, Li T, Guan L, Shi S, Zeng Q, Pei X, Chen L. Sonic hedgehog pathway is essential for maintenance of cancer stem-like cells in human gastric cancer. PLoS One. 2011 Mar 4;6(3):e17687. | Irrelevant study (Not relevant to CD 24) |
| 8 | Lorico A, Rappa G. Phenotypic heterogeneity of breast cancer stem cells. J Oncol. 2011;2011:135039. | Irrelevant study (Not relevant to gastric cancer) |
| 9 | Takaishi S, Okumura T, Tu S, Wang SS, Shibata W, Vigneshwaran R, Gordon SA, Shimada Y, Wang TC. Identification of gastric cancer stem cells using the cell surface marker CD44. Stem Cells. 2009 May;27(5):1006-20. | Irrelevant study (Not relevant to CD 24) |
| 10 | Hsu HC, Cheng W, Lai PL. Cloning and expression of a developmentally regulated transcript MXR7 in hepatocellular carcinoma: biological significance and temporospatial distribution. Cancer Res. 1997 Nov 15;57(22):5179-84. | Irrelevant study (Not relevant to CD 24 and gastric cancer) |
| 11 | Möller P, Matthaei-Maurer DU, Moldenhauer G. CD30(Ki-1) antigen expression in a subset of gastric mucosal plasma cells and in a primary gastric plasmacytoma. Am J Clin Pathol. 1989 Jan;91(1):18-23. | Irrelevant study (Not relevant to CD 24) |
| 12 | Mielke B, Möller P. Histomorphologic and immunophenotypic spectrum of primary gastro-intestinal B-cell lymphomas. Int J Cancer. 1991 Feb 1;47(3):334-43. | Irrelevant study (Not relevant to CD 24) |
| 13 | Dong XG, Niu JH, Zhao J, Mu TL, Yuan M. Expression and significaace of CXCL5 in precancerous lesion and gastric cancer. Journal of Nongken Medicine. 2009;30 (3):217-19. (Chinese) | Irrelevant study (Not relevant to CD 24) |
| 14 | Wang LL, He XS. Research progress of gastric carcinoma stem cells markers. Medical recapitulate. 2012,(6):851-854 (Chinese) | Article of review |
| 15 | Duan HR, Ling H. Advances in gastric cancer stem cells research. Journal of medical science in central south China.2012,(3):308-312. (Chinese) | Article of review |
| 16 | Cao L, Hu X, Zhang Y, Huang G. Significance of omental milky spots micrometastasis in screening gastric cancer stem cells and progenitor cells. Journal of surgery concepts and practice. 2011,(3):248-251. (Chinese) | Irrelevant study (Not relevant to CD 24) |
| 17 | Xu ZY, Cheng XD, Du YA, Huang L. Epithelial-mesenchymal transition in residual gastric cells after chemotherapy:An in vitro study. Chinese journal of clinical oncology. 2013,(18):1085-1088. (Chinese) | Irrelevant study (Not relevant to CD 24) |
| 18 | Lim SC, Oh SH. The role of CD24 in various human epithelial neoplasias. Pathol Res Pract. 2005;201(7):479-86. | Insufficient primary outcome data |
| 19 | Wang X, Jian T. The relationship between expression of CD24, microvascular density (MVD) and prognosis of gastric cancer. Shandong Mediacl Journal. 2008,(33):69. (Chinese) | Insufficient primary outcome data |
| 20 | Yong CS, Ou Yang CM, Chou YH, Liao CS, Lee CW, Lee CC. CD44/CD24 expression in recurrent gastric cancer: a retrospective analysis. BMC Gastroenterol. 2012 Jul 28;12:95. | Irrelevant study (Not relevant to the relationship between CD24 expression and gastric cancer clinicopathological parameters or prognosis) |
